# Immunological Characterization of Plant-Based HIV-1 Gag/Dgp41 Virus-Like Particles

**DOI:** 10.1371/journal.pone.0151842

**Published:** 2016-03-17

**Authors:** Sarah A. Kessans, Mark D. Linhart, Lydia R. Meador, Jacquelyn Kilbourne, Brenda G. Hogue, Petra Fromme, Nobuyuki Matoba, Tsafrir S. Mor

**Affiliations:** 1 School of Life Sciences, Arizona State University, Tempe, Arizona, United States of America; 2 Center for Infectious Diseases and Vaccinology, The Biodesign Institute, Arizona State University, Tempe, Arizona, United States of America; 3 Center for Applied Structural Discovery, The Biodesign Institute, Arizona State University, Tempe, Arizona, United States of America; 4 Department of Chemistry and Biochemistry, Arizona State University, Tempe, Arizona, United States of America; Mayo Clinic Arizona, UNITED STATES

## Abstract

It is widely anticipated that a prophylactic vaccine may be needed to control the HIV/AIDS epidemic worldwide. Despite over two decades of research, a vaccine against HIV-1 remains elusive, although a recent clinical trial has shown promising results. Recent studies have focused on highly conserved domains within HIV-1 such as the membrane proximal external region (MPER) of the envelope glycoprotein, gp41. MPER has been shown to play critical roles in mucosal transmission of HIV-1, though this peptide is poorly immunogenic on its own. Here we provide evidence that plant-produced HIV-1 enveloped virus-like particles (VLPs) consisting of Gag and a deconstructed form of gp41 comprising the MPER, transmembrane, and cytoplasmic domains (Dgp41) provides an effective platform to display MPER for use as an HIV vaccine candidate. Prime-boost strategies combining systemic and mucosal priming with systemic boosting using two different vaccine candidates (VLPs and CTB-MPR—a fusion of MPER and the B-subunit of cholera toxin) were investigated in BALB/c mice. Serum antibody responses against both the Gag and gp41 antigens were elicited when systemically primed with VLPs. These responses could be recalled following systemic boosting with VLPs. In addition, mucosal priming with VLPs allowed for a boosting response against Gag and gp41 when boosted with either candidate. Importantly, the VLPs also induced Gag-specific CD4 and CD8 T-cell responses. This report on the immunogenicity of plant-based Gag/Dgp41 VLPs may represent an important milestone on the road towards a broadly efficacious and inexpensive subunit vaccine against HIV-1.

## Introduction

The HIV-1 transmembrane subunit of the envelope protein (Env), gp41, contains the highly conserved membrane proximal external region, located just outside the lipid viral envelope (MPER, amino acids 661–683, [1]). The gp41 domain that encompasses the MPER and extends toward the C-terminal heptad repeat (residues 649–684, sometimes denoted as “MPR” but for simplicity we will refer to both as MPER, [2]) functions as a galactosyl-ceramide-binding lectin and is critical for mediating viral transcytosis across mucosal membranes [3] and other mucosal transmission routes [4, 5]. Both mucosal and systemic antibodies (Abs) raised against immunogens containing the MPER can block the transcytosis of HIV across the epithelial barrier [6, 7], similar to naturally occurring polyclonal mucosal IgAs found in the mucosal secretions of some highly exposed persistently seronegative (HEPS) individuals [8–11]. Revealingly, broadly neutralizing human monoclonal Abs (mAbs) such as 2F5, 4E10 and 10E8 also target this region [12–15]. The MPER, therefore, provides an important target for vaccine design, in addition to the widely-explored but highly-mutable surface subunit of Env (gp120, [16–19], reviewed in [20–22]).

The proximity of the MPER to the viral envelope is increasingly recognized as a major factor in the antigenicity and immunogenicity of the domain [23–25], suggesting that the presentation of the MPER in the context of a membrane, e.g. in virus-like particles (VLPs) may be of value. This notion and the recent success of prophylactic VLP-based vaccines such as those aimed at human papillomaviruses [26] provide the motivation for VLP-based vaccines against HIV-1. Gag, a polyprotein that gives rise to the main structural proteins of HIV-1, is both necessary and sufficient for the formation of enveloped VLPs [27, 28]. Gag contains the highest density of cytotoxic T-lymphocyte (CTL) epitopes of any HIV protein [29] and Gag-based VLPs are capable of inducing strong CTL responses without adjuvant [30]. CD8 T cell responses to Gag have been correlated with control of viral replication in infected individuals [31]. In addition, Gag VLPs can display HIV Env proteins on their surface in their native conformation [32], and these VLPs have been shown to induce both Env- and Gag-specific Abs and CTLs [33], making Gag VLPs attractive candidates as an HIV vaccine platform [34].

**Fig 1:**
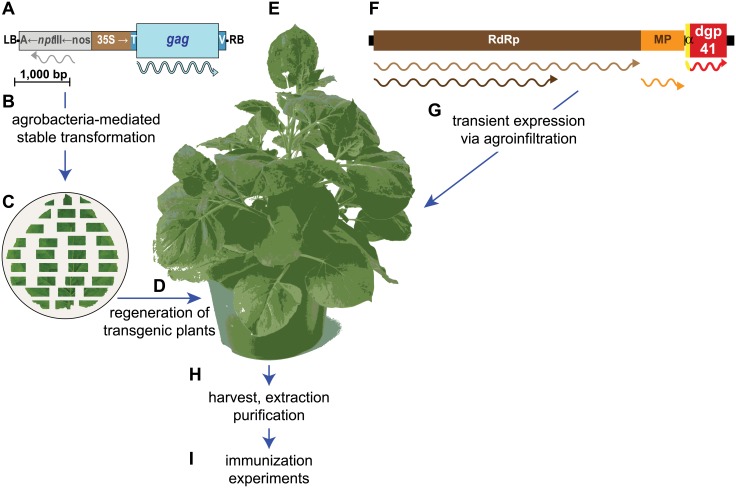
Plant-based strategy for the production of Gag-dgp41 VLPs. Plant-expression optimized synthetic gene encoding Gag was cloned into a T-DNA expression cassette of a binary vector **(A)**. *Agrobacterium tumefaciens* cells harboring the plasmid were used in the stable transformation of leaf explants from *Nicotiana benthamiana* **(B)**. Following co-cultivation **(C)** and selection on kanamycin, the transformed callus tissue was regenerated to obtain several lines of transgenic *N*. *benthamiana* plants **(D)** and the best expressing line was selected for further use **(E)**. Plant-expression optimized synthetic gene for the expression of the MPER-transmembrane and cytoplasmic domain of gp41 (dgp41) was cloned into the tobacco mosaic virus-based MagnICON vector system that recombine *in vivo* to yield a replicon that spreads from cell to cell **(F)**. Gag-expressing plants were infiltrated with agrobacteria that harbor the MagnICON vectors **(G)** and on peak accumulation day of the transiently-expressed dgp41, plant leaf material was harvested and homogenized **(H)**. VLPs were highly enriched by several purification steps **(I)**. Stable expression cassette: LB, left T-DNA border; A←*npt*III←nos, selectable marker consisting of the *nos* promoter, the *npt*III kanamycin resistance gene and the Agrobacterium gene 7 poly-A signal; 35S→T, cauliflower mosaic virus 35S promoter followed by the tobacco etch virus 5’ untranslated region, V, polyadenylation signal of soybean vegetative storage protein gene; RB, right T-DNA border. Transient expression replicon: RpRd, RNA-dependent RNA polymerase; MP, movement protein gene; α, barley alpha-amylase signal peptide. Wavy lines represent the translation products of the recombinant genes.

Plant-based production systems for biologics and vaccines lately reached several critical milestones gaining FDA approval for large-scale clinical trials and commercialization [35–39]. We previously reported that Gag VLPs displaying a deconstructed form of gp41 (Dgp41, comprising MPER, transmembrane, and cytoplasmic domains) could be produced in *Nicotiana benthamiana* plants (Fig 1) [40]. Here we report on immunization studies employing plant-based HIV-1 Gag/Dgp41 VLPs and demonstrate their immunogenicity.

## Materials and Methods

### Immunogens’ preparation

Highly-enriched plant-based VLPs were prepared by transiently expressing Dgp41 in Gag-expressing transgenic *N*. *benthamiana* plants and transiently expressing Dgp41 as previously described (see Fig 1 for expression and purification strategy) [40]. Quantitative immunoblots were use to quantify Gag and Dgp41 as previously described [40].

CTB-MPR is a fusion protein consisting of the HIV-1’s MPER fused to the carboxy-terminus of cholera toxin B-subunit (CTB, [41]). Expression of CTB-MPR in *Escherichia coli* and its purification previously described [41]. CTB-MPR preparation quality and yield were determined by Coomassie stained gels, quantitative immunoblots (using a pure standard), and the absorbance at 280 nm using ɛ = 2.1 mM^-1^ cm^-1^ [41]. Total protein was determined as previously described [42].

### Animal Care and Use

This study was reviewed and approved by the Arizona State University Institutional Animal Care and Use Committee (IACUC) under protocol number 11-1174R.

#### Housing and husbandry

All animals were housed in accordance with the American Association for Laboratory Animal Care (AALAC) standards. The animals are housed in Thoren ventilated racks that are HEPA filtered on both the supply and exhaust air on Irradiated Sani-Chip 7990. Environmental enrichment includes social housing and nestlets to encourage nesting activities. Animals are provided unlimited access to food and water. They are handled in accordance with the Animal Welfare Act and Institutional Animal Care and Use Committee (IACUC) regulations. Experiments involving animals were conducted in a facility fully accredited by the Association for Assessment and Accreditation of Laboratory Animal Care International (Unit #000765) and an assurance is on file with the Office for Laboratory Animal Welfare (#A3217-01). Experiments were planned and conducted utilizing the three R's (reduce, replace and refine), which included environmental enrichment, veterinary oversight, numbers reflecting statistical significance and the use of appropriate analgesics and anesthesia when appropriate.

#### Animal Monitoring

All animals are observed daily by trained DACT personnel for signs of illness or abnormal behavior by 10 AM. Training is provided by the Department of Animal Care and Technologies (DACT) veterinarians, experienced DACT personnel (i.e., Lead Technologists, Supervisors). Personnel performing the daily observations report sick or injured animals to the DACT vet team. During regular work hours, personnel can contact the veterinary team in person, by phone, or via email using a veterinary team distribution list. Outside of working hours, a veterinary team member carries a dedicated on-call cell phone. A call list with contact numbers for supervisory and veterinary personnel is posted in each animal facility.

The mice are monitored for activity level that may be indicative of illness such as hypoactivity (abnormally low}, hyperactivity (abnormally high), lethargy, restlessness. They are monitored for behavioral signs such as vocalization, self-trauma, aggressiveness, isolation from cage mates, or ataxia. They are monitored for changes to their appearance such as unkempt or greasy fur, porphyrin staining around eyes and nostrils, hunched posture, pale mucous membranes, pale paws, soiled anogenital area, labored breathing, weight loss, dehydration or diarrhea. Animals that show obvious signs of illness are either removed from the study and treated if it is relevant, or euthanized immediately, based upon the recommendation of the veterinary team.

#### Euthanasia

Mice used in this study were euthanized by CO2 asphyxiation, which is consistent with the most recent recommendations of the American Veterinary Medical Association [43]. Cervical dislocation or secondary thoracotomy was used as a subsequent secondary measure. No animals died during the experiments. One mouse belonging to the control group (see below) was found to be in lateral recumbency and experiencing labored breathing. Since this was the day before the final bleeding, the mouse was euthanized immediately. Gross necropsy was performed and revealed slightly enlarged kidneys, gas in the gastrointestinal tract (indicative of not eating), and dehydration. This was not deemed as a result of the study as the mouse was in our control group. This was one day before scheduled termination so there was no significant impact on our study.

### Systemic immunization

**Fig 2:**
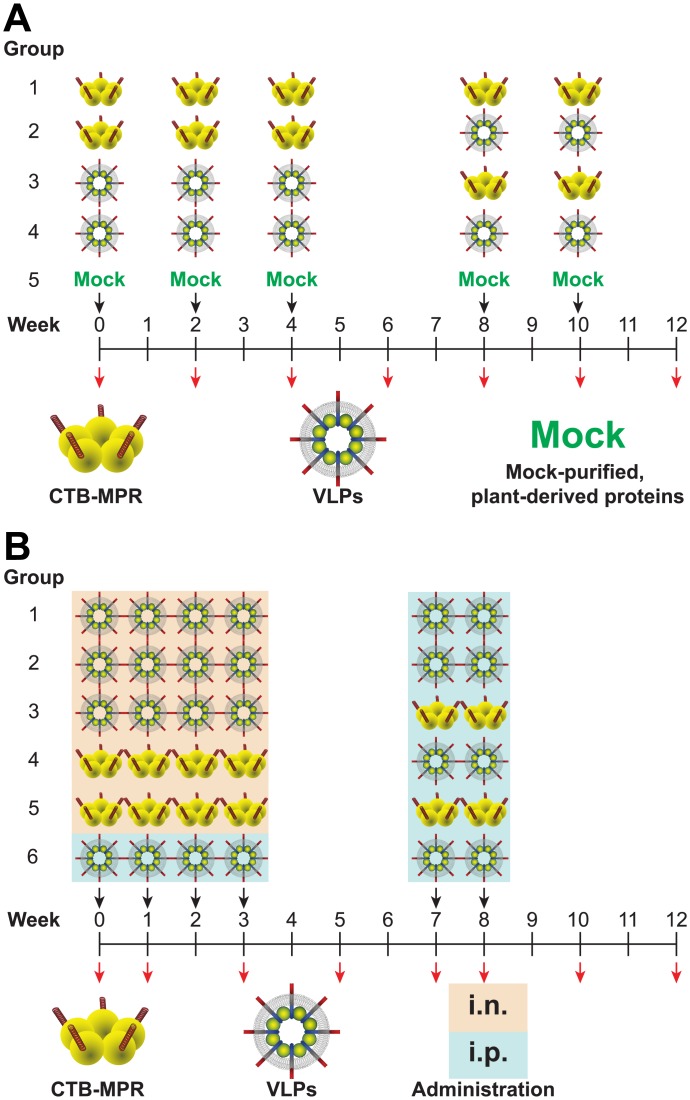
**(A) Immunization scheme for systemic immunization**. Mice were given three priming immunizations on Weeks 0, 2, and 4, and two boosting immunizations on Weeks 8 and 10 (black arrows). Mice in Group 1 were primed and boosted with CTB-MPR; mice in Group 2 were primed with CTB-MPR and boosted with VLPs; mice in Group 3 were primed with VLPs and boosted with CTB-MPR, mice in Group 4 were primed and boosted with VLPs, and mice in Group 5 were mock immunized with plant derived proteins as described in the Methods. Blood samples were taken on Weeks 0, 2, 4, 8, 10, and 12 (red arrows). **(B) Immunization scheme for mucosal immunization**. Mice were given four priming immunizations on Weeks 0, 1, 2, and 3, and two boosting immunizations on Weeks 7 and 8 (black arrows). Mice in Group 1 were mucosally primed with VLPs and murabutide and systemically boosted with VLPs; mice in Group 2 were mucosally primed with VLPs and CT and systemically boosted with VLPs; mice in Group 3 were mucosally primed with VLPs and CT and systemically boosted with CTB-MPRs; mice in Group 4 were mucosally primed with CTB-MPR and CT and systemically boosted with VLPs; mice in Group 5 were mucosally primed with CTB-MPR and CT and systemically boosted with CTB-MPR, and mice in Group 6 were systemically primed and boosted with VLPs. Blood, fecal, and vaginal samples were taken on Weeks 0, 1, 3, 5, 7, 8, 10, and 12 (red arrows).

The immunization samples was prepared by diluting the concentrated protein preparations into PBS supplemented with Ribi Adjuvant (Sigma-Aldrich, final concentration of 2% oil as per manufacturer’s instructions). Female BALB/c mice (6-wk old, Charles River) were immunized intraperitoneally (i.p.) with 200 μL of the immunization sample containing either CTB-MPR (3.5 μg ≡ 1.2 μg MPER ≡ 0.2 nmol MPER), VLP preparation (111 μg total protein containing 4.8 μg dgp41 and 4.8 μg Gag, equivalent to 0.2 nmol and 0.1 nmol, respectively), or a negative control sample (100 μg total protein, mock-purified in the same manner as the VLP proteins). Four experimental groups (n = 8) were given either VLP or CTB-MPR during each of three priming immunizations, and were then given either VLP or CTB-MPR during each of two boosting immunizations (Fig 2A). A fifth group was immunized with the negative control sample under the same regimen. Retro-orbital vein blood samples were taken as indicated. Serum was prepared following clotting and centrifugation and kept at –80°C until further use.

### Mucosal immunization

For intranasal (i.n.) administration, immunization cocktails were made by mixing the indicated proteins with PBS. Each dose (20 μl total, 10 μl per nostril) contained either CTB-MPR (35 μg ≡ 12 μg MPER ≡ 2 nmol MPER) with cholera toxin (CT, 1 μg, List Biological Laboratories), VLP preparation (1.1 mg total protein containing 48 μg dgp41 and 48 μg Gag, equivalent to 2 nmol and 1 nmol, respectively) with either CT (1 μg), or murabutide (200 μg, InvivoGen). Female BALB/c mice (6-wk old, Charles River, n = 8 per group) were given four i.n. priming immunizations (Fig 2B). Group 1 received VLPs+murabutide, Groups 2 and 3 received VLPs+CT, Groups 4 and 5 received CTB-MPR+CT. Group 6, serving as a control, was immunized i.p. with VLPs, as described above. Mice in all groups were given two i.p. boosting immunizations with either VLPs (4.8 μg dgp41 and 4.8 μg Gag, Groups 1, 2, 4 and 6) or CTB-MPR (3.5 μg), Groups 3 and 5, Fig 2B), administered with Ribi Adjuvant as per the systemic trial above. Serum, vaginal secretions, and fecal pellets were collected from all mice as indicated. Abs were extracted from fecal pellets by soaking five pellets (~50 mg) in PBS containing 0.02% Na-azide (500 μL) for 30 min at 4°C with occasional vortex and clarification by centrifugation (14,000 ×*g*, 10 min). Vaginal secretions were collected by lavage using PBS (100 μL) with a blunt-tipped syringe needle. Serum, fecal Abs, and vaginal lavages were kept at -80°C.

### Antibody Titer Assays

ELISA plates were coated with 20 μg of streptavidin (Sigma-Aldrich) and 2 μg of biotinylated MPR peptide (for detection of anti-MPER Abs) or 1 μg of p24-CTA2 (for detection of anti-Gag Abs). Binding of the MPR peptide through its N-terminal biotin should maximize accessibility the accessibility of the peptide to interactions with cognate antibodies [41]. The wells were overlaid with a threefold serial dilution of serum, vaginal secretions or fecal samples (starting with 1:50, 1:5 or 1:2, respectively) in PBS containing 0.5% Tween 20 and 5% dry milk and the procedure was continued as previously described [41]. Endpoint titers were determined as the reciprocal of the dilution factor of sample giving background levels of OD_490_. Statistical analysis of data was by the Kruskal-Wallis test followed by Dunn’s Multiple Comparison test.

### IFN-γ ELISPOT Assay

Splenocytes were prepared from pooled harvested spleens on Week 12. Interferon-gamma (IFN-γ) Enzyme-Linked Immunosorbent Spot Assay (ELISPOT) responses were measured using a mouse IFN-γ set (BD Biosciences). Threefold serially diluted triplicates of splenocytes (starting at 1 x 10^6^ splenocytes/well) were applied to the plates in a final volume of 200 μl RPMI 1640 culture medium (with 10% heat-inactivated fetal bovine serum, 100 U/mL penicillin, 100 μg streptomycin). The peptides AAMQMLKDTINEEAA (corresponding to the GagCD8 epitope, from HIV-1 Consensus C Gag (15-mer) Peptides, Cat#8118, NIH AIDS Reagent Program) and SNPPVPVGDIYKRWI/VPVGDIYKRWIILGL (corresponding to the GagCD4 epitope, from HIV-1 Consensus C Gag (15-mer) Peptides, Cat#8118, NIH AIDS Reagent Program) were used as stimuli in the assay at 5 μg/mL. Reactions without peptide served as background controls. Reactions were allowed to proceed for 30 h at 37°C in a humidified 5% CO_2_ atmosphere. Spots were detected with the detection antibody, developed with 3-amino-9-ethyl-carbazole (BD Biosciences), and analyzed using a CTL ImmunoSpot plate reader and counting software (Cellular Technology Ltd). For each group of mice, the number of background spots in the absence of peptide was subtracted from the average of the triplicate values in order to determine the number of peptide-relevant spots.

## Results

### Serum anti-Gag responses to systemic immunizations

**Fig 3:**
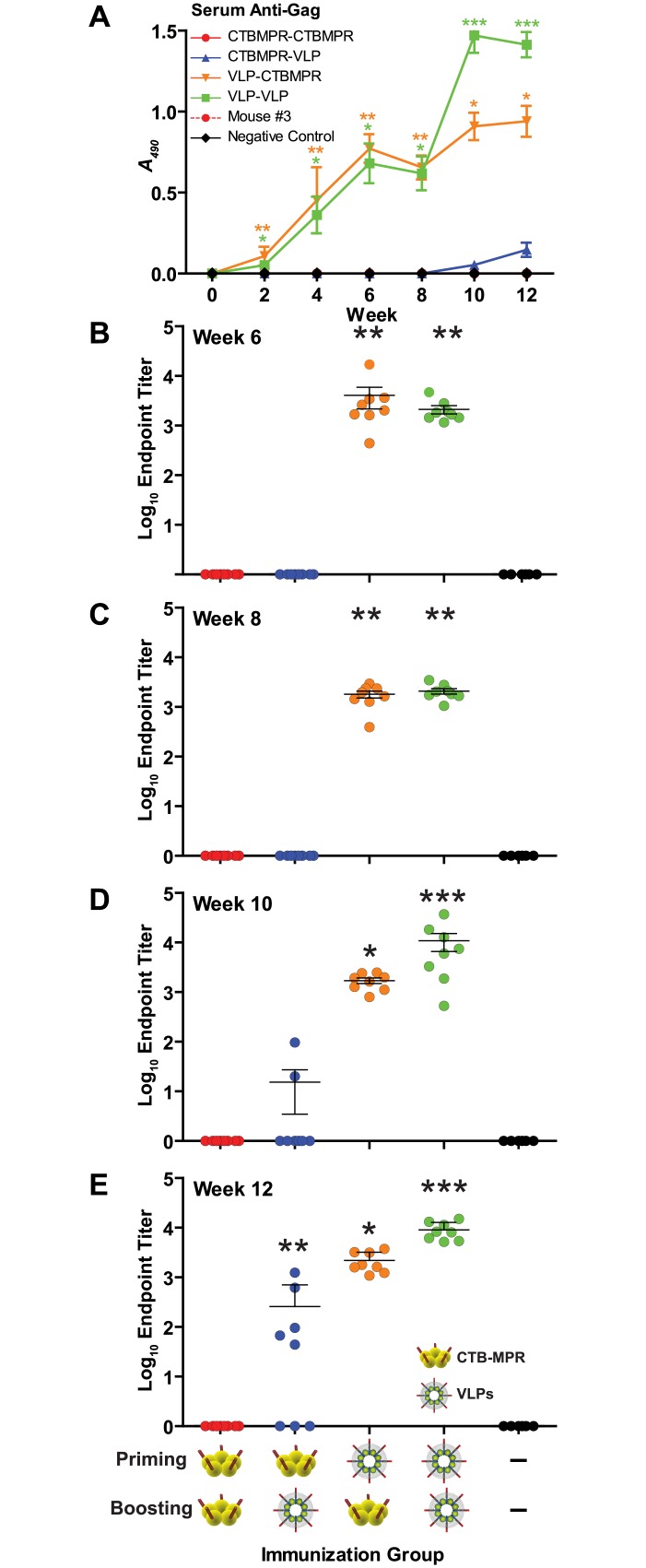
Serum anti-p24 (anti-Gag) responses. **(A)** Mice were immunized as per Fig 2A. Serum samples were diluted 1:50 and IgGs were detected by direct ELISA on indicated weeks. Shown are average net OD490 values (mean +/- SEM). Also shown are Ab endpoint titers at response peaks after priming (**B,** Week 6); before first boost (**C,** Week 8), after first boost (**D,** Week 10), and after second boost (**E,** Week 12). Symbols indicate statistical significance (compared to week zero within the group) evaluated by Kruskal-Wallis test and Dunn’s Multiple Comparison test: * p < 0.05, ** p < 0.01, *** p < 0.001.

Anti-p24 Abs were quantified after the third priming immunization (Week 6), before the first boost (Week 8), after the first boost (Week 10), and after the second boost (Week 12, Fig 3). In all mice (16/16) primed with VLPs, significant Ab titers (p < 0.01, in comparison to the negative control group) were elicited after priming and remained steady until the first boost (Fig 3B–3E). The VLP-primed animals were then split into two groups and boosted with either CTB-MPR or VLPs. CTB-MPR-boosted mice retained significant titers of anti-p24 Abs through the end of the trial, despite the fact that the mice did not receive any further Gag protein. Boosting with VLPs resulted in increased antibody titers, with final antibody titers reaching extremely significant (p< 0.001) values over the negative control mice. The majority of mice (5/8) primed with CTB-MPR and boosted with VLPs developed anti-p24 Ab responses at similar titers to VLP-primed animals with two doses. All of these data demonstrate that plant-produced VLPs can elicit robust, long-lived Ab responses against the Gag protein, which are detectable at high titers at least eight weeks after the final VLP immunization (our final time point) for VLP/CTB-MPR-immunized animals.

### Serum anti-MPER responses to systemic immunizations

**Fig 4:**
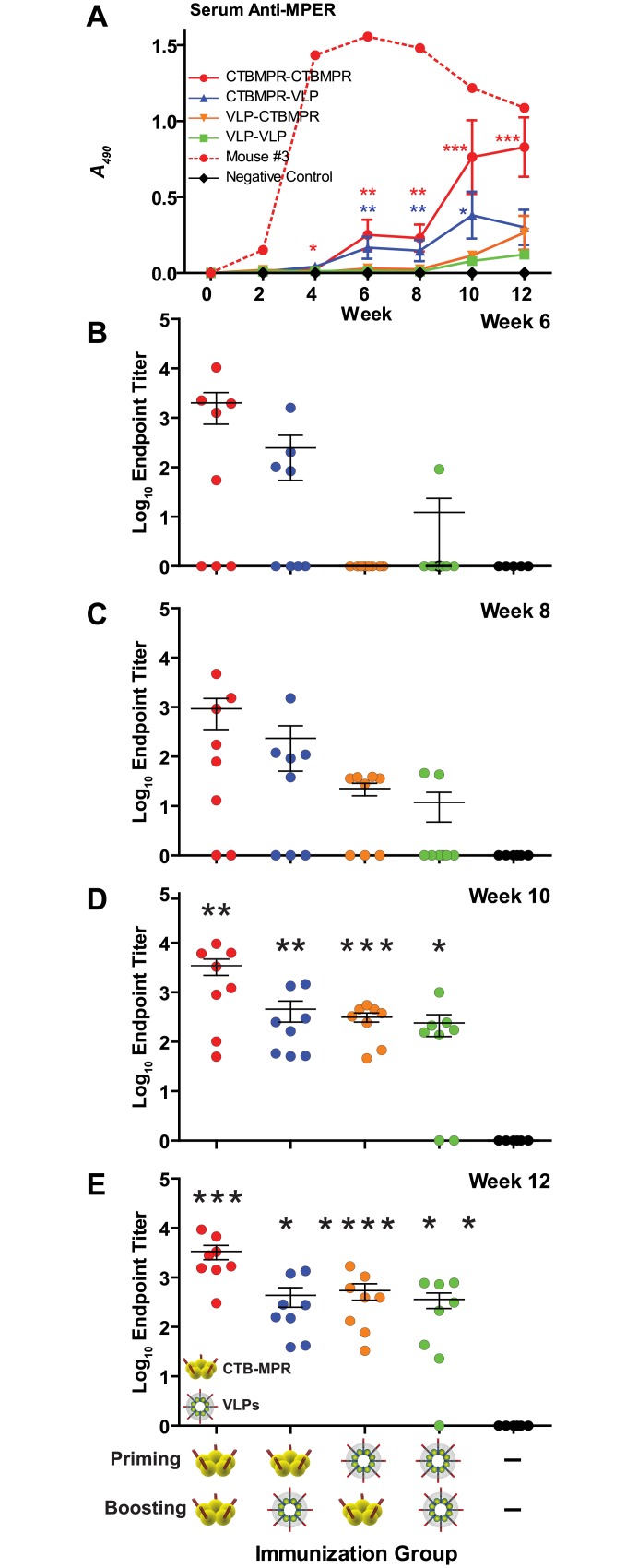
Serum anti-MPER IgG levels. **(A)** Mice were immunized as per Fig 2A. Serum samples were diluted 1:50 and IgGs were detected by direct ELISA on indicated weeks. Shown are average net OD490 values (mean +/- SEM). Mouse #3 (from CTBMPR-CTBMPR group) responded significantly higher than other mice from this group and was considered an outlier, and antibody response from this mouse is shown by itself. Also shown are Ab endpoint titers at response peaks after priming (**B,** Week 6); before first boost (**C,** Week 8), after first boost (**D,** Week 10), and after second boost (**E,** Week 12). Symbols indicate statistical significance as compared to week zero within the group evaluated by Kruskal-Wallis test and Dunn’s Multiple Comparison test: * p < 0.05, ** p < 0.01, *** p < 0.001.

Most (11/16) mice primed with CTB-MPR responded to the MPER moiety before the second boost (Fig 4). One of these mice (#3 from the CTB-MPR-primed and boosted group) responded remarkably better than the rest of the mice (shown on its own, Fig 4). VLP priming induced detectable anti-MPER Abs in 7/16 mice. All animals boosted with CTB-MPR, regardless of priming group, displayed significant anti-MPER antibody titers (p < 0.001 and p < 0.01, respectively) after the second boost. In 7/8 mice primed with VLPs and boosted with VLPs, significant (p < 0.05) titers were elicited after the first boost, which steadily increased following the second boost. All four experimental groups elicited statistically significant Ab levels following the second boost. However, CTB-MPR appears to be a slightly more potent inducer of anti-MPER Abs, though this difference was not statistically significant.

### Serum anti-Gag antibody response to mucosal immunization

Because HIV-1’s main mode of transmission mode is the crossing of mucosal barriers in the female genital tract and the lower gastro-intestinal tract, we further tested the ability of plant-derived VLPs to stimulate mucosal responses. To this end, immunogens were used to test the effectiveness of intranasal priming with systemic boosting of plant-produced VLPs, CTB-MPR, or a combination of both.

**Fig 5:**
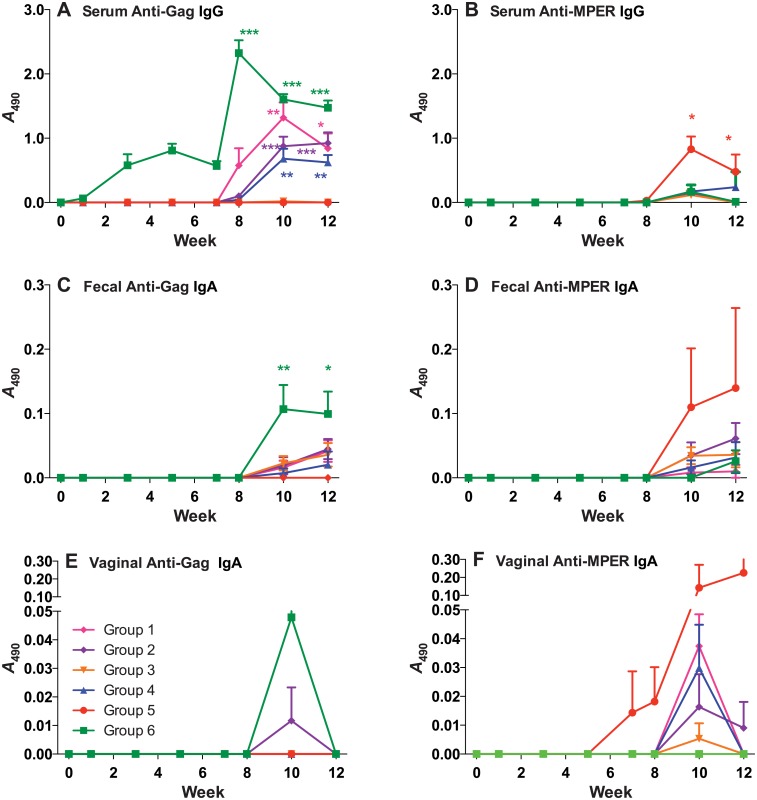
Serum IgG and mucosal IgA responses across all weeks of mucosal immunization. Anti-Gag (**A, C, E**) and Anti MPER (**B, D, F**) serum IgG (**A and B**), fecal mucosal IgA (**C and D**), and vaginal mucosal IgA (**E and F**) were assayed in samples obtained on weeks 0,1, 3, 5, 7, 8, 10, and 12. Time points are averages of all mice in each group. Symbols indicate statistical significance as compared to week zero within the group evaluated by Kruskal-Wallis test and Dunn’s Multiple Comparison test: * p < 0.05, ** p < 0.01, *** p < 0.001.

Anti-p24 Abs were not detected in any of the mucosally-primed mice prior to boosting (Fig 5), although systemically-primed mice displayed anti-p24 serum IgG titers consistent with the first immunization trial (see above) with 6/8 mice responding to Gag following the first priming immunization. The majority of mice (30/32) in all groups that were i.p.-boosted with VLPs responded to the Gag protein by eliciting very high Ab titers (Fig 5). These results confirm the effectiveness of using VLPs in a systemic prime/boost regimen to elicit a response against Gag, and also suggest the effectiveness of mucosal priming with systemic boosting to elicit a similar response.

### Serum anti-MPER antibody response to mucosal immunization

**Fig 6:**
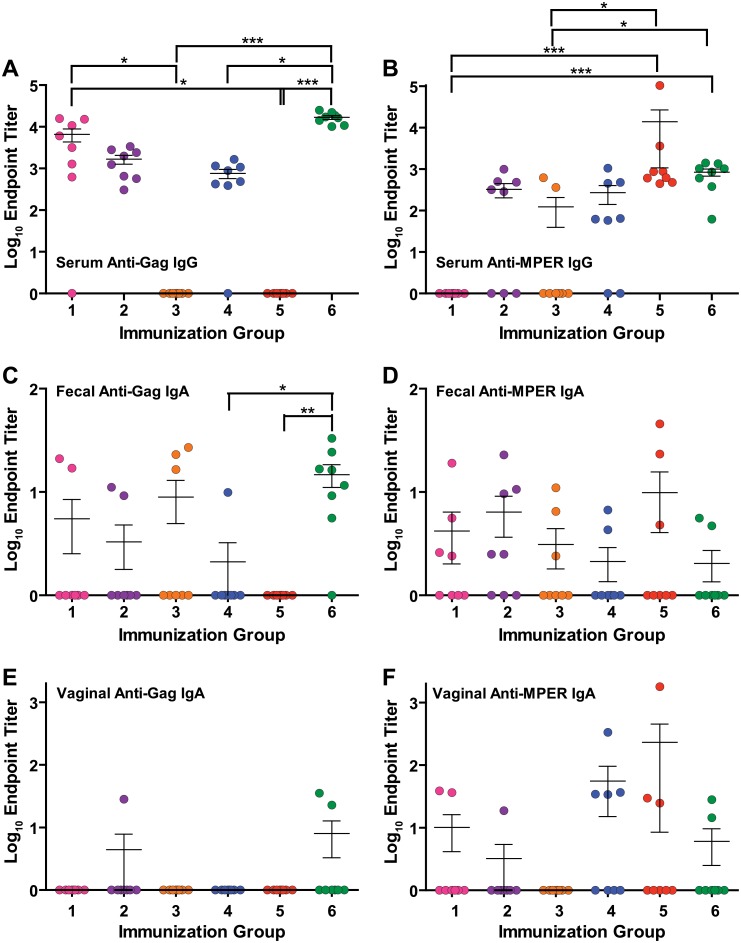
Endpoint titers. Post-boosting (Week 10) antibody endpoint titers against Gag (**A, C, E**) and MPER (**B, D, F**)in serum (IgG, **A and B**), fecal (secretory IgA, **C and D**), and vaginal (secretory IgA, **E and F**) samples. Symbols indicate statistical significance as compared to week zero within the group evaluated by Kruskal-Wallis test and Dunn’s Multiple Comparison test: * p < 0.05, ** p < 0.01, *** p < 0.001.

As was the case of the anti-p24 response, anti-MPR serum Abs were below the detection limit in all mucosally-primed mice prior to boosting immunizations (Fig 5). In accordance with the systemic immunization experiment, no significant Ab titers against MPER were raised in the systemically-primed mice prior to boosting, either. Systemic boosting with CTB-MPR after mucosal priming with the same immunogen (Group 5) elicited significant (p < 0.001, Fig 6) levels of Abs as compared to naïve mice, consistent with previous studies [6]. Interestingly, mucosal priming with VLPs and boosting with either VLPs or CTB-MPR (Groups 1, 2, and 3) elicited only marginal levels of Abs, and the same was observed for mucosal priming with CTB-MPR and boosting with VLPs. Although all the mice responded in Group 6 (systemically primed and boosted with VLPs), the levels of Abs were lower in this group than in the CTB-MPR primed and boosted group (Group 5). However, we note that the differences between the two groups were not statistically significant.

### Fecal anti-Gag and anti-MPER responses to mucosal immunizations

Mucosal response against Gag was limited in all groups either primed or boosted with VLPs, although 6/8 mice were positive for IgAs in fecal samples in Group 6 (systemically primed and boosted with VLPs). Responses from Groups 1, 2, and 3 (mucosally primed with VLPs) were all statistically similar. Additionally, mucosal VLP priming induced slightly higher Ab titers with a greater number of responders as compared to those primed with CTB-MPR (Groups 4 and 5, Fig 6). While the difference was not statistically significant, the trend suggests that mucosal priming with VLPs might assist in eliciting a mucosal response against Gag, though this response is not as strong as that elicited with systemic priming with VLPs.

Similar to the anti-p24 mucosal response, we could not detect any fecal anti-MPER IgAs in any of the treatment groups until after the final immunization. Two weeks after the final boost, low anti-MPER IgA responses could be detected in 12/48 mice. The two responders in Group 5 had two of the highest responses, but no group reached statistically significant responses in comparison to naïve mice (Figs 4 and 5). Mice in Groups 2, 3, and 4 had similar responses. Group 1 had a single responder while all mice in Group 6 had fecal anti-MPER responses below the limit of detection. The results of MPER response in fecal samples are inconclusive, but suggest that mice mucosally primed and systemically boosted with CTB-MPR can elicit a moderate IgA response in fecal samples, while systemic priming and boosting with VLPs is largely ineffective in eliciting a mucosal anti-MPER response in mice.

### Vaginal anti-Gag and anti-MPER response in mucosal immunizations

Anti-p24 IgA Abs could not be detected in any vaginal secretions of the mice prior to boosting immunizations, and only three mice (one from Group 2 and two from Group 6) responded two weeks after the second boost.

In accordance with low levels of anti-MPER Abs in fecal samples, only 12/48 mice from all groups responded to MPER in vaginal secretions. Once again, two mice in Group 5 (the same mice with higher anti-MPER response in fecal samples) had two of the highest overall responses, while vaginal anti-MPER responses from other groups were minimal or undetectable. Consistent with fecal results, no anti-MPER Abs were detected in vaginal secretions from mice in Group 6. Overall, the results of anti-MPER response in vaginal secretions are congruent with results of anti-MPER response in fecal samples. These results suggest that while systemic priming and boosting with VLPs can be successful in eliciting antibody responses against Gag in serum and at mucosal sites, mucosal VLP priming may not be nearly as effective in this case, with minimal responses seen to Dgp41. Mucosal immunization elicits the highest antibody responses to gp41 at all sites in Group 5, which is primed and boosted with CTB-MPR.

### IFN-γ ELISPOT Assay

**Fig 7:**
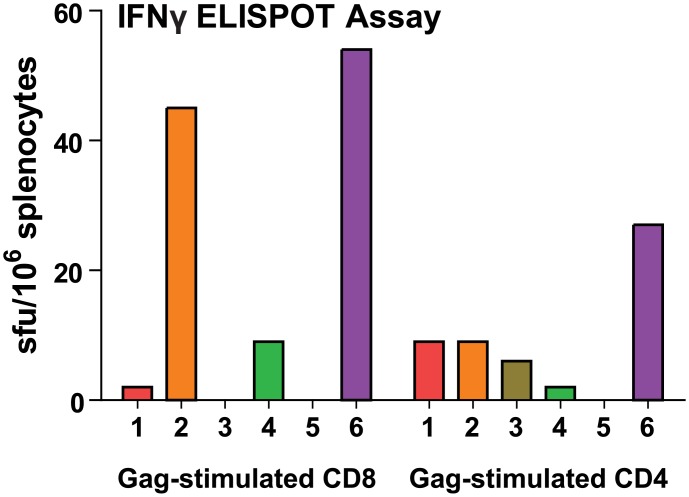
IFN-γ ELISPOT assay. Splenocytes were harvested, isolated, and pooled from mice in each group and incubated with or without peptides corresponding to the Gag CD8 or GagCD4epitopes. Data are averages of triplicate values, with number of background spots from wells containing no peptide subtracted from this average.

In addition to the humoral and mucosal immune responses, the cellular responses against peptides corresponding to Gag-specific CD4 and CD8 immunodominant epitopes were also assayed two weeks following the final immunization for the vaccination experiment described in Fig 3. Mice primed (either mucosally or systemically) and boosted with Gag/Dgp41 VLPs had expression of IFN-y at levels over 40sfu/10^6^ cells in response to the CD8 epitope, suggesting a high CTL response against Gag in these groups (Fig 7). Overall, CD4 responses were lower with the highest response being mice primed and boosted systemically with VLPs at over 20 sfu per million splenocytes (Group 6, Fig 7). Minimal, but detectable, CD4 responses were seen with all groups primed with VLPs regardless of boosting regimen (Groups 1–3, Fig 6). In general, animals primed and boosted with VLPs induced higher T cell responses than those with mixed regimens (compare groups 2 and 6 to 1, 3, and 4). As expected, mice primed and boosted with CTB-MPR did not respond to either peptide (Group 5, Fig 7). These results suggest that both CD8 and CD4 T-cell responses against Gag were elicited with the VLPs with the most successful regimen being systemic administration.

## Discussion

The MPER domain of gp41 in HIV-1, which plays multiple roles in early stages of the viral life-cycle, is targeted by Abs with strong, broad and diverse antiviral activities including transcytosis blockade (e.g. [3, 7, 9, 41, 44]), neutralization (e.g. [8, 15, 45, 46]), and Ab-dependent cell cytotoxicity [47]. Based on these findings, the MPER deservedly became the focus of intensifying vaccine development research.

Because the MPER is poorly immunogenic on its own, a platform with which to present the peptide is required and various carrier proteins have been tested (e.g. [48, 49]). Our lab has tested the immunogenicity of multiple MPER fusion proteins which utilized CTB [6, 7, 41], *Yersinia pestis* antigens [50], and hepatitis B core antigen (Cherni, Matoba, and Mor, unpublished). These studies demonstrated the success of heterologous prime-boost strategies with multiple routes of immunization and more than one vaccine construct in eliciting transcytosis-blocking Abs. However, an optimal presentation platform to display the MPER peptide that would ensure a uniformly strong immune response against MPER, while overcoming the immune-dominance of the carrier proteins, is still elusive.

The ability of the MPER to elicit Abs with antiviral activities is context dependent. In particular the proximity to the membrane plays a significant role. For example, recently it was demonstrated using a knock-in mouse model that 2F5 Abs could be elicited to very high titers *in vivo* only when MPER was displayed in the context of a membrane [51]. Similarly, the MPER peptide (“P1”) attached to virosomes was shown to elicit transcytosis-blocking Abs in both serum and mucosal sites in macaques (where their elicitation correlated with protection from infection [52]) and humans [19]. Within the context of an intact Env, the MPER is available to bind neutralizing mAbs exclusively during the pre-hairpin intermediate stage of the infection process. Outside of this window, binding is hindered because the MPER is either sterically-obscured (e.g. on the surface of a virion), or strongly interacting with other gp41 domains [53]. These conformational considerations, among others (such as clonal anergy, [54]) contribute to the low propensity for induction of 2F5-like Abs during the acute phase of HIV-1 infection [53].

We previously demonstrated that plants can efficiently make HIV-1 enveloped VLPs consisting of p55^GAG^ and a membrane-anchored stripped-down Env that we call “deconstructed gp41” [40]. Devoid of the immunodominant gp120 and the N-terminal regions of gp41, we hypothesized that such VLPs would present the MPER in a conformation that approximate the pre-fusion intermediate stage [2]. Interestingly, recent structural analyses of a similar gp41 construct containing the MPER and the transmembrane domains of gp41 (MPR-TM) reveal that indeed, the neutralizing epitopes of 4E10 and 2F5 are accessible to these monoclonal antibodies when the protein is presented in a non-denatured trimeric state [2, 55]. The antibodies bind to the MPR-TM with sub-nanomolar affinities as determined by competitive ELISA and surface plasmon resonance [2, 55].

The plant-derived VLPs incorporate the Gag protein and the deconstructed envelope protein Dgp41 at Gag:Dgp41 ratios that vary slightly between 1:1 [40] and 2:1 (here). Previous studies by others aimed at producing recombinant VLPs reported lower levels of Env protein incorporation. For example, Hammonds and co-workers reported much higher Gag:Env ratios in VLPs produced in human cell lines (varying between 25:1 to 50:1) or through baculovirus infection of insect cells (10:1) [56]. The presence of more dgp41 molecules on the surface of plant-derived VLPs as compared to other recombinant VLPs is immunologically advantageous.

Here we tested the hypothesis that such plant-derived VLPs could serve to elicit immune responses against the presented antigens. As shown above, HIV-1 Gag/Dgp41 VLPs produced in *N*. *benthamiana* were able to elicit substantial titers of Abs against both Gag and gp41 epitopes through a heterologous platform prime-boost regimens in conjunction with CTB-MPR. Intraperitoneally-administered VLPs were also effective as immunogens on their own (Figs 2–5) and significant levels of Abs were raised against the MPER after boosting with VLPs. Our ELISA detection system is not likely to enable us to detect potential conformational Abs and therefore potentially underestimates the humoral response to the VLPs. Although mucosal priming immunizations were not sufficient to elicit Abs against Gag or gp41, such responses were shown for animals that subsequently were boosted with systemically-delivered plant-produced VLPs (Fig 6). In conjunction with the VLP induction of strong Ab responses, we detected Gag-specific CD4 and CD8 T-cell responses, which were the highest in groups primed and boosted with VLPs regardless of immunization route (Fig 7).

The use of plants as production platforms for candidate subunit vaccines is now well established, achieving important milestones in the last couple of years including winning FDA approval for use in humans [39, 57]. As was recently demonstrated by stepping up plant production of a promising anti-Ebola virus mAb cocktail [39, 58, 59], plants offer a highly competitive alternative in terms of costs, scale-up and safety to traditional production of vaccines such as fermentation (yeast and animal cell cultures), or eggs. In particular, plants were shown to express VLPs such as those of Hepatitis B virus [60, 61], Norwalk virus [62, 63], influenza [64], and even HIV-1 [65, 66].

Only few studies were published presenting immunogenicity data for plant-based HIV-1 full-length Gag or membrane-anchored gp41. Our co-expression system [40] was the first to produce enough full-length p55^Gag^ together with Dgp41 to conduct a study like the one we describe here (Gag yield is ~ 22 mg/kg fresh leaf material). However, plant-expressed derivatives of Gag (i.e. p24, p17, and p41) were tested and were shown to elicit anti-Gag Abs as well as CTL responses [65, 67–70]. In addition, several studies demonstrated success in eliciting immunological responses against plant-based MPER constructs [71–75], however none were membrane anchored. While we acknowledge that further investigation is necessary to elicit a more robust and durable anti-MPER Ab response and demonstrate its protective efficacy in HIV-1 challenge models, our results here offer evidence that plant-based Gag/Dgp41 VLPs can induce relevant Ab and T cell responses and forms the basis for the development of an effective component for future HIV-1 prime/boost immunization trials.

## References

[1] Montero M, van Houten NE, Wang X, Scott JK. The membrane-proximal external region of the human immunodeficiency virus type 1 envelope: dominant site of antibody neutralization and target for vaccine design. Microbiol Mol Biol Rev. 2008;72(1):54–84. 10.1128/MMBR.00020-07 18322034 PMC2268283

[2] Gong Z, Kessans SA, Song L, Dorner K, Lee HH, Meador LR, et al Recombinant expression, purification, and biophysical characterization of the transmembrane and membrane proximal domains of HIV-1 gp41. Protein Sci. 2014;23(11):1607–18. 10.1002/pro.2540 25155369 PMC4241111

[3] Alfsen A, Iniguez P, Bouguyon E, Bomsel M. Secretory IgA Specific for a Conserved Epitope on gp41 Envelope Glycoprotein Inhibits Epithelial Transcytosis of HIV-1. J Immunol. 2001;166(10):6257–65. . 11342649 10.4049/jimmunol.166.10.6257

[4] Sagar M, Akiyama H, Etemad B, Ramirez N, Freitas I, Gummuluru S. Transmembrane Domain Membrane Proximal External Region but Not Surface Unit-Directed Broadly Neutralizing HIV-1 Antibodies Can Restrict Dendritic Cell-Mediated HIV-1 Trans-infection. J Infect Dis. 2012;205(8):1248–57. Epub 2012/03/08. jis183 [pii] 10.1093/infdis/jis183 22396600 PMC3308909

[5] Magerus-Chatinet A, Yu H, Garcia S, Ducloux E, Terris B, Bomsel M. Galactosyl ceramide expressed on dendritic cells can mediate HIV-1 transfer from monocyte derived dendritic cells to autologous T cells. Virology. 2007;362(1):67–74. . 17234232 10.1016/j.virol.2006.11.035

[6] Matoba N, Geyer BC, Kilbourne J, Alfsen A, Bomsel M, Mor TS. Humoral immune responses by prime-boost heterologous route immunizations with CTB-MPR(649–684), a mucosal subunit HIV/AIDS vaccine candidate. Vaccine. 2006;24:5047–55. . 16621185 10.1016/j.vaccine.2006.03.045

[7] Matoba N, Magerus A, Geyer BC, Zhang Y, Muralidharan M, Alfsen A, et al A mucosally targeted subunit vaccine candidate eliciting HIV-1 transcytosis-blocking Abs. Proc Natl Acad Sci U S A. 2004;101(37):13584–9. . 15347807 10.1073/pnas.0405297101 PMC518798

[8] Broliden K, Hinkula J, Devito C, Kiama P, Kimani J, Trabbatoni D, et al Functional HIV-1 specific IgA antibodies in HIV-1 exposed, persistently IgG seronegative female sex workers. Immunol Lett. 2001;79(1–2):29–36. . 11595287 10.1016/s0165-2478(01)00263-2

[9] Devito C, Broliden K, Kaul R, Svensson L, Johansen K, Kiama P, et al Mucosal and plasma IgA from HIV-1-exposed uninfected individuals inhibit HIV-1 transcytosis across human epithelial cells. J Immunol. 2000;165(9):5170–6. . 11046049 10.4049/jimmunol.165.9.5170

[10] Kaul R, Plummer F, Clerici M, Bomsel M, Lopalco L, Broliden K. Mucosal IgA in exposed, uninfected subjects: evidence for a role in protection against HIV infection. Aids. 2001;15(3):431–2. . 11273233 10.1097/00002030-200102160-00026

[11] Benjelloun F, Dawood R, Urcuqui-Inchima S, Rochereau N, Chanut B, Verrier B, et al Secretory IgA specific for MPER can protect from HIV-1 infection in vitro. AIDS. 2013;27(12):1992–5. Epub 2013/05/23. 10.1097/QAD.0b013e3283632ea1 . 23696073

[12] Zwick MB, Jensen R, Church S, Wang M, Stiegler G, Kunert R, et al Anti-human immunodeficiency virus type 1 (HIV-1) antibodies 2F5 and 4E10 require surprisingly few crucial residues in the membrane-proximal external region of glycoprotein gp41 to neutralize HIV-1. J Virol. 2005;79(2):1252–61. 10.1128/JVI.79.2.1252-1261.2005 15613352 PMC538539

[13] Hessell AJ, Rakasz EG, Tehrani DM, Huber M, Weisgrau KL, Landucci G, et al Broadly neutralizing monoclonal antibodies 2F5 and 4E10 directed against the human immunodeficiency virus type 1 gp41 membrane-proximal external region protect against mucosal challenge by simian-human immunodeficiency virus SHIVBa-L. Journal of virology. 2010;84(3):1302–13. Epub 2009/11/13. 10.1128/JVI.01272-09 19906907 PMC2812338

[14] Kwong PD, Mascola JR. Human antibodies that neutralize HIV-1: identification, structures, and B cell ontogenies. Immunity. 2012;37(3):412–25. Epub 2012/09/25. 10.1016/j.immuni.2012.08.012 . 22999947 PMC4706166

[15] Huang J, Ofek G, Laub L, Louder MK, Doria-Rose NA, Longo NS, et al Broad and potent neutralization of HIV-1 by a gp41-specific human antibody. Nature. 2012;491(7424):406–12. Epub 2012/11/16. 10.1038/nature11544 . 23151583 PMC4854285

[16] Bouvin-Pley M, Morgand M, Meyer L, Goujard C, Moreau A, Mouquet H, et al Drift of the HIV-1 envelope glycoprotein gp120 toward increased neutralization resistance over the course of the epidemic: a comprehensive study using the most potent and broadly neutralizing monoclonal antibodies. J Virol. 2014;88(23):13910–7. 10.1128/JVI.02083-14 25231299 PMC4248973

[17] Dennison SM, Sutherland LL, Jaeger FH, Anasti KM, Parks R, Stewart S, et al Induction of antibodies in rhesus macaques that recognize a fusion-intermediate conformation of HIV-1 gp41. PLoS One. 2011;6(11):e27824 10.1371/journal.pone.0027824 22140469 PMC3227606

[18] Pastori C, Tudor D, Diomede L, Drillet AS, Jegerlehner A, Rohn TA, et al Virus like particle based strategy to elicit HIV-protective antibodies to the alpha-helic regions of gp41. Virology. 2012;431(1–2):1–11. Epub 2012/06/05. 10.1016/j.virol.2012.05.005 . 22658900

[19] Leroux-Roels G, Maes C, Clement F, van Engelenburg F, van den Dobbelsteen M, Adler M, et al Randomized Phase I: Safety, Immunogenicity and Mucosal Antiviral Activity in Young Healthy Women Vaccinated with HIV-1 Gp41 P1 Peptide on Virosomes. PloS one. 2013;8(2):e55438 Epub 2013/02/26. 10.1371/journal.pone.0055438 23437055 PMC3577797

[20] Burton DR, Ahmed R, Barouch DH, Butera ST, Crotty S, Godzik A, et al A Blueprint for HIV Vaccine Discovery. Cell host & microbe. 2012;12(4):396–407. Epub 2012/10/23. 10.1016/j.chom.2012.09.008 23084910 PMC3513329

[21] Koff WC, Burton DR, Johnson PR, Walker BD, King CR, Nabel GJ, et al Accelerating next-generation vaccine development for global disease prevention. Science. 2013;340(6136):1232910 10.1126/science.1232910 23723240 PMC4026248

[22] Verkoczy L, Kelsoe G, Haynes BF. HIV-1 envelope gp41 broadly neutralizing antibodies: hurdles for vaccine development. PLoS Pathog. 2014;10(5):e1004073 10.1371/journal.ppat.1004073 24853821 PMC4031215

[23] Ivankin A, Apellaniz B, Gidalevitz D, Nieva JL. Mechanism of membrane perturbation by the HIV-1 gp41 membrane-proximal external region and its modulation by cholesterol. Biochimica et biophysica acta. 2012;1818(11):2521–8. Epub 2012/06/14. 10.1016/j.bbamem.2012.06.002 . 22692008 PMC8796276

[24] Montero M, Gulzar N, Klaric KA, Donald JE, Lepik C, Wu S, et al Neutralizing epitopes in the membrane-proximal external region of HIV-1 gp41 are influenced by the transmembrane domain and the plasma membrane. J Virol. 2012;86(6):2930–41. Epub 2012/01/13. JVI.06349-11 [pii] 10.1128/JVI.06349-11 22238313 PMC3302331

[25] Ofek G, Zirkle B, Yang Y, Zhu Z, McKee K, Zhang B, et al Structural basis for HIV-1 neutralization by 2F5-like antibodies m66 and m66.6. J Virol. 2014;88(5):2426–41. 10.1128/JVI.02837-13 24335316 PMC3958054

[26] Tumban E, Peabody J, Peabody DS, Chackerian B. A universal virus-like particle-based vaccine for human papillomavirus: longevity of protection and role of endogenous and exogenous adjuvants. Vaccine. 2013;31(41):4647–54. 10.1016/j.vaccine.2013.07.052 23933337 PMC3785330

[27] Balasubramaniam M, Freed EO. New insights into HIV assembly and trafficking. Physiology (Bethesda). 2011;26(4):236–51. Epub 2011/08/16. 10.1152/physiol.00051.2010 . 21841072 PMC3467973

[28] Gheysen D, Jacobs E, de Foresta F, Thiriart C, Francotte M, Thines D, et al Assembly and release of HIV-1 precursor Pr55gag virus-like particles from recombinant baculovirus-infected insect cells. Cell. 1989;59(1):103–12. . 2676191 10.1016/0092-8674(89)90873-8

[29] Addo MM, Yu XG, Rathod A, Cohen D, Eldridge RL, Strick D, et al Comprehensive epitope analysis of human immunodeficiency virus type 1 (HIV-1)-specific T-cell responses directed against the entire expressed HIV-1 genome demonstrate broadly directed responses, but no correlation to viral load. J Virol. 2003;77(3):2081–92. . 12525643 10.1128/JVI.77.3.2081-2092.2003 PMC140965

[30] Doan LX, Li M, Chen C, Yao Q. Virus-like particles as HIV-1 vaccines. Rev Med Virol. 2005;15(2):75–88. . 15484204 10.1002/rmv.449

[31] Kiepiela P, Ngumbela K, Thobakgale C, Ramduth D, Honeyborne I, Moodley E, et al CD8+ T-cell responses to different HIV proteins have discordant associations with viral load. Nat Med. 2007;13(1):46–53. 10.1038/nm1520 . 17173051

[32] Hicar MD, Chen X, Briney B, Hammonds J, Wang JJ, Kalams S, et al Pseudovirion particles bearing native HIV envelope trimers facilitate a novel method for generating human neutralizing monoclonal antibodies against HIV. J Acquir Immune Defic Syndr. 2010;54(3):223–35. 10.1097/QAI.0b013e3181dc98a3 20531016 PMC2930513

[33] Montefiori DC, Safrit JT, Lydy SL, Barry AP, Bilska M, Vo HT, et al Induction of neutralizing antibodies and gag-specific cellular immune responses to an R5 primary isolate of human immunodeficiency virus type 1 in rhesus macaques. J Virol. 2001;75(13):5879–90. 10.1128/JVI.75.13.5879-5890.2001 11390589 PMC114303

[34] Williamson AL, Rybicki EP. Justification for the inclusion of Gag in HIV vaccine candidates. Expert Rev Vaccines. 2015:1–14. 10.1586/14760584.2016.1129904 . 26645951

[35] Zimran A, Gonzalez-Rodriguez DE, Abrahamov A, Elstein D, Paz A, Brill-Almon E, et al Safety and efficacy of two dose levels of taliglucerase alfa in pediatric patients with Gaucher disease. Blood Cells Mol Dis. 2015;54(1):9–16. 10.1016/j.bcmd.2014.10.002 . 25453586

[36] Zhang Y, Li D, Jin X, Huang Z. Fighting Ebola with ZMapp: spotlight on plant-made antibody. Science China Life sciences. 2014;57(10):987–8. 10.1007/s11427-014-4746-7 . 25218825

[37] Lyon GM, Mehta AK, Varkey JB, Brantly K, Plyler L, McElroy AK, et al Clinical care of two patients with Ebola virus disease in the United States. N Engl J Med. 2014;371(25):2402–9. 10.1056/NEJMoa1409838 . 25390460

[38] Kuehn BM. As Ebola Epidemic Begins to Slow, Trials of Drugs and Vaccines Speed Up. JAMA. 2015 10.1001/jama.2015.0942 . 25671623

[39] Rybicki EP. Plant-based vaccines against viruses. Virology journal. 2014;11(1):205 10.1186/s12985-014-0205-0 25465382 PMC4264547

[40] Kessans SA, Linhart MD, Matoba N, Mor T. Biological and biochemical characterization of HIV-1 Gag/dgp41 virus-like particles expressed in Nicotiana benthamiana. Plant Biotechnology Journal. 2013;11(6):681–90. 10.1111/pbi.12058 23506331 PMC3688661

[41] Matoba N, Griffin TA, Mittman M, Doran JD, Alfsen A, Montefiori DC, et al Transcytosis-blocking abs elicited by an oligomeric immunogen based on the membrane proximal region of HIV-1 gp41 target non-neutralizing epitopes. Curr HIV Res. 2008;6(3):218–29. . 18473785 10.2174/157016208784324994 PMC2744741

[42] Mor TS, Sternfeld M, Soreq H, Arntzen CJ, Mason HS. Expression of recombinant human acetylcholinesterase in transgenic tomato plants. Biotechnol Bioeng. 2001;75(3):259–66. . 11590598 10.1002/bit.10012

[43] Leary S, Underwood W, Anthony R, Cartner S, Corey D, Grandin T, et al AVMA guidelines for the euthanasia of animals: 2013 edition (Available at: https://www.avma.org/KB/Policies/Documents/euthanasia.pdf). Schaumburg, IL: American Veterinary Medical Association; 2013 102 p.

[44] Shen R, Drelichman ER, Bimczok D, Ochsenbauer C, Kappes JC, Cannon JA, et al GP41-specific antibody blocks cell-free HIV type 1 transcytosis through human rectal mucosa and model colonic epithelium. J Immunol. 2010;184(7):3648–55. Epub 2010/03/09. 10.4049/jimmunol.0903346 20208001 PMC3731077

[45] Zwick MB, Labrijn AF, Wang M, Spenlehauer C, Saphire EO, Binley JM, et al Broadly neutralizing antibodies targeted to the membrane-proximal external region of human immunodeficiency virus type 1 glycoprotein gp41. J Virol. 2001;75(22):10892–905. 10.1128/JVI.75.22.10892-10905.2001 11602729 PMC114669

[46] Tudor D, Derrien M, Diomede L, Drillet AS, Houimel M, Moog C, et al HIV-1 gp41-specific monoclonal mucosal IgAs derived from highly exposed but IgG-seronegative individuals block HIV-1 epithelial transcytosis and neutralize CD4(+) cell infection: an IgA gene and functional analysis. Mucosal Immunol. 2009;2(5):412–26. Epub 2009/07/10. mi200989 [pii] 10.1038/mi.2009.89 . 19587640

[47] Tudor D, Bomsel M. The broadly neutralizing HIV-1 IgG 2F5 elicits gp41-specific antibody-dependent cell cytotoxicity in a FcgammaRI-dependent manner. AIDS. 2011;25(6):751–9. 10.1097/QAD.0b013e32834507bd . 21330910

[48] Wang J, Xu L, Tong P, Chen YH. Mucosal antibodies induced by tandem repeat of 2F5 epitope block transcytosis of HIV-1. Vaccine. 2011;29(47):8542–8. Epub 2011/09/24. 10.1016/j.vaccine.2011.09.032 . 21939723

[49] Zhou M, Kostoula I, Brill B, Panou E, Sakarellos-Daitsiotis M, Dietrich U. Prime boost vaccination approaches with different conjugates of a new HIV-1 gp41 epitope encompassing the membrane proximal external region induce neutralizing antibodies in mice. Vaccine. 2012;30(11):1911–6. Epub 2012/01/25. S0264-410X(12)00041-2 [pii] 10.1016/j.vaccine.2012.01.026 . 22269872

[50] Matoba N, Shah NR, Mor TS. Humoral immunogenicity of an HIV-1 envelope residue 649–684 membrane-proximal region peptide fused to the plague antigen F1-V. Vaccine. 2011;29(34):5584–90. Epub 2011/06/23. S0264-410X(11)00871-1 [pii] 10.1016/j.vaccine.2011.06.007 21693158 PMC3152316

[51] Verkoczy L, Chen Y, Zhang J, Bouton-Verville H, Newman A, Lockwood B, et al Induction of HIV-1 broad neutralizing antibodies in 2F5 knock-in mice: selection against membrane proximal external region-associated autoreactivity limits T-dependent responses. Journal of immunology. 2013;191(5):2538–50. Epub 2013/08/07. 10.4049/jimmunol.1300971 23918977 PMC3870053

[52] Bomsel M, Tudor D, Drillet AS, Alfsen A, Ganor Y, Roger MG, et al Immunization with HIV-1 gp41 Subunit Virosomes Induces Mucosal Antibodies Protecting Nonhuman Primates against Vaginal SHIV Challenges. Immunity. 2011;34(2):269–80. Epub 2011/02/15. S1074-7613(11)00036-7 [pii] 10.1016/j.immuni.2011.01.015 . 21315623

[53] Frey G, Peng H, Rits-Volloch S, Morelli M, Cheng Y, Chen B. A fusion-intermediate state of HIV-1 gp41 targeted by broadly neutralizing antibodies. P Natl Acad Sci USA. 2008;105(10):3739–44. Epub 2008/03/07. 10.1073/pnas.0800255105 18322015 PMC2268799

[54] Haynes BF, Verkoczy L. AIDS/HIV. Host controls of HIV neutralizing antibodies. Science. 2014;344(6184):588–9. 10.1126/science.1254990 24812389 PMC4162091

[55] Gong Z, Martin-Garcia JM, Daskalova SM, Craciunescu FM, Song L, Dorner K, et al Biophysical Characterization of a Vaccine Candidate against HIV-1: The Transmembrane and Membrane Proximal Domains of HIV-1 gp41 as a Maltose Binding Protein Fusion. PLoS One. 2015;10(8):e0136507 10.1371/journal.pone.0136507 26295457 PMC4546420

[56] Hammonds J, Chen X, Zhang X, Lee F, Spearman P. Advances in methods for the production, purification, and characterization of HIV-1 Gag-Env pseudovirion vaccines. Vaccine. 2007;25(47):8036–48. 10.1016/j.vaccine.2007.09.016 . 17936444

[57] Rybicki EP. Plant-made vaccines for humans and animals. Plant Biotechnol J. 2010;8(5):620–37. Epub 2010/03/18. PBI507 [pii] 10.1111/j.1467-7652.2010.00507.x . 20233333 PMC7167690

[58] Qiu X, Wong G, Audet J, Bello A, Fernando L, Alimonti JB, et al Reversion of advanced Ebola virus disease in nonhuman primates with ZMapp. Nature. 2014;514(7520):47–53. 10.1038/nature13777 25171469 PMC4214273

[59] McCarthy M. US signs contract with ZMapp maker to accelerate development of the Ebola drug. Bmj. 2014;349:g5488 10.1136/bmj.g5488 . 25189475

[60] Thanavala Y, Mahoney M, Pal S, Scott A, Richter L, Natarajan N, et al Immunogenicity in humans of an edible vaccine for hepatitis B. Proc Natl Acad Sci U S A. 2005;102(9):3378–82. . 15728371 10.1073/pnas.0409899102 PMC549291

[61] Huang Z, Chen Q, Hjelm B, Arntzen C, Mason H. A DNA replicon system for rapid high-level production of virus-like particles in plants. Biotechnology and bioengineering. 2009;103(4):706–14. Epub 2009/03/25. 10.1002/bit.22299 19309755 PMC2704498

[62] Santi L, Batchelor L, Huang Z, Hjelm B, Kilbourne J, Arntzen CJ, et al An efficient plant viral expression system generating orally immunogenic Norwalk virus-like particles. Vaccine. 2008;26(15):1846–54. Epub 2008/03/08. 10.1016/j.vaccine.2008.01.053 18325641 PMC2744496

[63] Zhang X, Buehner NA, Hutson AM, Estes MK, Mason HS. Tomato is a highly effective vehicle for expression and oral immunization with Norwalk virus capsid protein. Plant biotechnology journal. 2006;4(4):419–32. Epub 2006/12/21. 10.1111/j.1467-7652.2006.00191.x . 17177807

[64] D'Aoust MA, Couture MM, Charland N, Trepanier S, Landry N, Ors F, et al The production of hemagglutinin-based virus-like particles in plants: a rapid, efficient and safe response to pandemic influenza. Plant biotechnology journal. 2010;8(5):607–19. Epub 2010/03/05. 10.1111/j.1467-7652.2009.00496.x . 20199612

[65] Meyers A, Chakauya E, Shephard E, Tanzer FL, Maclean J, Lynch A, et al Expression of HIV-1 antigens in plants as potential subunit vaccines. BMC Biotechnol. 2008;8:53 10.1186/1472-6750-8-53 18573204 PMC2443125

[66] Scotti N, Alagna F, Ferraiolo E, Formisano G, Sannino L, Buonaguro L, et al High-level expression of the HIV-1 Pr55gag polyprotein in transgenic tobacco chloroplasts. Planta. 2009;229(5):1109–22. 10.1007/s00425-009-0898-2 19234717

[67] Guetard D, Greco R, Cervantes Gonzalez M, Celli S, Kostrzak A, Langlade-Demoyen P, et al Immunogenicity and tolerance following HIV-1/HBV plant-based oral vaccine administration. Vaccine. 2008;26(35):4477–85. Epub 2008/07/08. S0264-410X(08)00806-2 [pii] 10.1016/j.vaccine.2008.06.059 . 18601967

[68] Perez-Filgueira DM, Brayfield BP, Phiri S, Borca MV, Wood C, Morris TJ. Preserved antigenicity of HIV-1 p24 produced and purified in high yields from plants inoculated with a tobacco mosaic virus (TMV)-derived vector. Journal of virological methods. 2004;121(2):201–8. Epub 2004/09/24. 10.1016/j.jviromet.2004.06.022 . 15381357

[69] Gonzalez-Rabade N, McGowan EG, Zhou F, McCabe MS, Bock R, Dix PJ, et al Immunogenicity of chloroplast-derived HIV-1 p24 and a p24-Nef fusion protein following subcutaneous and oral administration in mice. Plant biotechnology journal. 2011;9(6):629–38. Epub 2011/03/30. 10.1111/j.1467-7652.2011.00609.x . 21443546

[70] Lindh I, Kalbina I, Thulin S, Scherbak N, Savenstrand H, Brave A, et al Feeding of mice with Arabidopsis thaliana expressing the HIV-1 subtype C p24 antigen gives rise to systemic immune responses. Apmis. 2008;116(11):985–94. Epub 2009/01/10. 10.1111/j.1600-0463.2008.00900.x . 19132995

[71] Durrani Z, McInerney TL, McLain L, Jones T, Bellaby T, Brennan FR, et al Intranasal immunization with a plant virus expressing a peptide from HIV-1 gp41 stimulates better mucosal and systemic HIV-1-specific IgA and IgG than oral immunization. J Immunol Methods. 1998;220(1–2):93–103. . 9839930 10.1016/s0022-1759(98)00145-8

[72] Marusic C, Rizza P, Lattanzi L, Mancini C, Spada M, Belardelli F, et al Chimeric plant virus particles as immunogens for inducing murine and human immune responses against human immunodeficiency virus type 1. J Virol. 2001;75(18):8434–9. . 11507188 10.1128/JVI.75.18.8434-8439.2001 PMC115088

[73] Matoba N, Kajiura H, Cherni I, Doran JD, Alfsen A, Bomsel M, et al Biochemical and immunological characterization of the plant-derived candidate HIV-1 mucosal vaccine CTB MPR_649 684_. Plant Biotechnol J. 2009;7(2):129–45. 10.1111/j.1467-7652.2008.00381.x 19037902

[74] McInerney TL, Brennan FR, Jones TD, Dimmock NJ. Analysis of the ability of five adjuvants to enhance immune responses to a chimeric plant virus displaying an HIV-1 peptide. Vaccine. 1999;17(11–12):1359–68. . 10195771 10.1016/s0264-410x(98)00388-0

[75] McLain L, Durrani Z, Wisniewski LA, Porta C, Lomonossoff GP, Dimmock NJ. Stimulation of neutralizing antibodies to human immunodeficiency virus type 1 in three strains of mice immunized with a 22 amino acid peptide of gp41 expressed on the surface of a plant virus. Vaccine. 1996;14(8):799–810. . 8817828 10.1016/0264-410x(95)00229-t

